# Active and Mild Chronic Necrotizing Crescentic Glomerulonephritis With Severe Medullary Angiitis in an Elderly Male Patient: A Case Report

**DOI:** 10.7759/cureus.87036

**Published:** 2025-06-30

**Authors:** Harpreet S Dosanjh, Ariel Ahl, Muhammad Durrani, Garo Kalfayan, Arian Gower

**Affiliations:** 1 Internal Medicine, Los Robles Regional Medical Center, Thousand Oaks, USA

**Keywords:** acute kidney injury, anca-associated vasculitis, necrotizing and crescentic glomerulonephritis, neph, pauci-immune crescentic glomerulonephritis, rheum

## Abstract

Pauci-immune crescentic glomerulonephritis (GN) is a rapidly progressive form of GN typically associated with antineutrophil cytoplasmic antibody (ANCA)-associated vasculitis (AAV) and may present with rare but severe histologic findings such as medullary angiitis. We describe a 69-year-old male patient with no prior medical history who presented with worsening renal function following a recent hospitalization for what is thought to be pneumonia and presumed prerenal azotemia. On readmission, he was found to have an elevated serum creatinine of 5.42 mg/dL, significant hematuria and proteinuria, elevated inflammatory markers, and normocytic anemia. Further evaluation revealed positive P-ANCA at a 1:640 titer and elevated serum IgG. A renal biopsy confirmed the diagnosis of pauci-immune necrotizing crescentic GN with both acute and chronic features, severe medullary angiitis, and arterial nephrosclerosis. The patient was treated with intravenous pulse methylprednisolone, followed by rituximab and avacopan, resulting in significant improvement in renal function without the need for dialysis. This case highlights the diagnostic challenge of distinguishing intrinsic renal disease from prerenal azotemia in the setting of acute illness and underscores the importance of considering AAV in patients presenting with unexplained acute kidney injury and systemic inflammatory markers. Medullary angiitis, although infrequently reported, may be associated with more severe renal disease and should be recognized as a potential marker of disease severity. Early biopsy and prompt initiation of immunosuppressive therapy can lead to substantial renal recovery, even in the presence of chronic histologic damage.

## Introduction

Pauci-immune crescentic glomerulonephritis (GN) is a rapidly progressive form of GN often associated with antineutrophil cytoplasmic antibody (ANCA)-related vasculitis, including conditions like microscopic polyangiitis (MPA) and, less frequently, eosinophilic granulomatosis with polyangiitis. Among the ANCA subtypes, perinuclear ANCA (p-ANCA) typically directed against myeloperoxidase is strongly linked to MPA and plays a critical role in disease pathogenesis. p-ANCA-associated vasculitis often presents with systemic manifestations, including constitutional symptoms, pulmonary involvement, and renal impairment. Renal involvement may progress rapidly, manifesting as hematuria, proteinuria, and acute kidney injury, frequently culminating in crescentic GN. Histopathologically, this condition is marked by necrotizing glomerular inflammation with crescent formation and little to no immunoglobulin deposition, a finding that distinguishes it from immune complex-mediated diseases. Early recognition and aggressive immunosuppressive therapy including corticosteroids, rituximab, and novel agents such as avacopan are critical in preventing irreversible renal damage and reducing morbidity and mortality. This case highlights the importance of early recognition and intervention in ANCA-associated GN, a condition that, if left untreated, can rapidly progress to end-stage renal disease and increased risk of mortality [1].

## Case presentation

A 69-year-old patient with no significant past medical history presented to the hospital from their primary care doctor for outpatient labs that showed an elevated serum creatinine. To note, three weeks prior, the patient was hospitalized for one day with pneumonia and an acute kidney injury, thought to be prerenal azotemia. 

**Figure 1:**
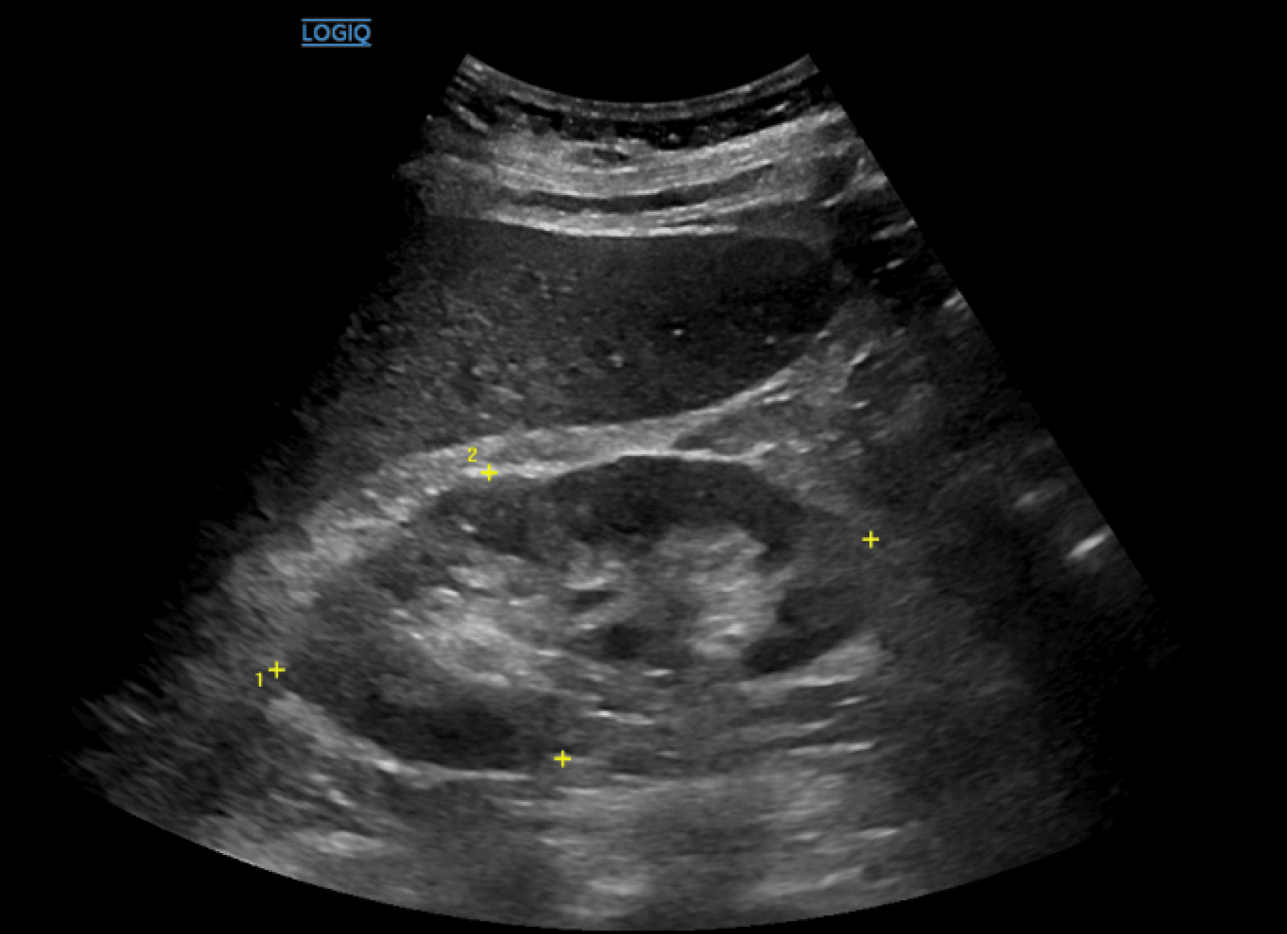
The central echolucent (dark) area extends beyond the renal sinus and appears somewhat bulging outside the renal hilum, which is typical of an extrarenal pelvis

On examination, the patient appeared in no apparent distress and only complained of minimal fatigue. Initial laboratory tests revealed blood urea nitrogen of 78 mg/dL, serum creatinine of 5.42 mg/dL, phosphorus of 6.2 mg/dL, and potassium of 5.8 mg/dL. The patient was also noted to have elevated inflammatory markers (C-reactive protein of 29.1 mg/L and erythrocyte sedimentation rate of 92 ng/mL) and normocytic anemia with a hemoglobin of 9.4 g/dL. Lab data and reference points are listed in Table 1. Urine studies were collected, but ultimately, a calculated fractional excretion of sodium was indeterminate, being 1.4%. Urinalysis indicated leukocyte esterase of 250 Leu/uL, 1+ protein, 3+ blood, RBC count of 27/HPF, and WBC of 24/HPF. A renal ultrasound (Figure 1) revealed mildly prominent right renal pelvis versus extrarenal pelvis, with no hydronephrosis. 

**Table 1:** Laboratory values and reference points

Test/Parameter	Patient Result	Reference Range
Blood Urea Nitrogen (BUN)	78 mg/dL	7–20 mg/dL
Serum Creatinine	5.42 mg/dL	0.6–1.3 mg/dL
Phosphorus	6.2 mg/dL	2.5–4.5 mg/dL
Potassium	5.8 mmol/L	3.5–5.0 mmol/L
Hemoglobin	9.4 g/dL	13.5–17.5 g/dL
CRP	29.1 mg/L	<5 mg/L
ESR	92 mm/hr	<20 mm/hr
Urinalysis: Leukocyte Esterase	250 Leu/uL	Negative
Urinalysis: Protein	1+	Negative
Urinalysis: Blood	3+	Negative
RBCs in Urine	27/HPF	0–3/HPF
WBCs in Urine	24/HPF	0–5/HPF
FE Na	Indeterminate 1.4%	1–2%
Microalbumin/Creatinine	453	<30
C3/C4	Normal	Normal
IgG	1806 mg/dL	700–1600 mg/dL
Total Globulin	4.4 g/dL	2.0–3.5 g/dL
P-ANCA	1:640	Negative

During hospitalization, the patient was started on intravenous fluids, and nephrology was consulted. Further studies, including hepatitis panel (ABC), rheumatoid factor, ANA, and HIV, were found to be negative. Complement C3/C4 was found to be within normal limits. Protein electrophoresis and serum immunofixation indicated an increased total globulin of 4.4 g/dL and increased IgG of 1806 mg/dL. Further urine studies demonstrated a severely increased microalbumin to creatinine ratio of 453 mg/g. During the hospitalization course, renal biopsy was obtained, and during that time, anti-neutrophil cytoplasmic antibody testing indicated findings of p-ANCA at a 1:640 titer. The patient was started on pulse steroids of methylprednisolone 500 mg IV q24h x3 days and showed improvement in kidney function. Kidney biopsy revealed active and mild chronic necrotizing crescentic glomerulonephritis with severe medullary angiitis, along with arterial nephrosclerosis. The patient was started on rituximab and avacopan to decrease the amount of corticosteroid usage. The patient’s renal function improved without the need to initiate hemodialysis, with creatinine down trending from 5.42 to 2.80 and increased estimated glomerular filtration rate. The patient was ultimately discharged with a prolonged course of steroids of prednisone for two weeks and follow-up for a possibly longer course with atovaquone (pneumocystis prevention) and pantoprazole (stress ulcer prevention) for prophylaxis, rituximab, avacopan, and outpatient follow-up with nephrology.

## Discussion

Pauci-immune crescentic GN presents as a severe form of rapidly progressive GN often associated with AAV [2]. This condition is characterized by inflammation and necrosis of small vessels, which can lead to multiorgan failure [3]. In renal AAV-associated pauci-immune GN, the hallmark is noted to be a necrotizing and crescentic glomerular lesion [3,4].

Renal biopsy remains the gold standard for diagnosis and key for differentiation between different vasculitis-induced GN [5]. The histopathologic finding of necrotizing crescentic GN with medullary angiitis and arterial nephrosclerosis underscores the severity of vascular inflammation and chronicity of disease in this patient. Histopathologic features of medullary angiitis are described as interstitial hemorrhage and necrosis in the medulla with associated polymorphonuclear leukocyte infiltration and presence of karyorrhectic debris [6]. Although medullary angiitis is rarely reported, it is identified in approximately 9.5% cases of AAV and associated with severe renal impairment and multisystem disease [7]. This patient's degree of global and segmental sclerosis (29% and 8%, respectively), along with arterial nephrosclerosis, reflects chronic changes that contribute to his renal impairment and are important for prognostication [8].

Early recognition and introduction of immunosuppressive therapy are critical in AAV to prevent progression to end-stage kidney disease without the use of long-term corticosteroids. This patient responded favorably to an induction regimen of pulse methylprednisolone, followed by rituximab and avacopan. Rituximab, a monoclonal anti-CD20 antibody, has emerged as a first-line treatment for induction therapy in AAV, showing non-inferiority to cyclophosphamide in clinical trials [9]. Avacopan, a C5a receptor antagonist, was shown to improve kidney function and reduce glucocorticoid exposure, as shown in the ADVOCATE trial [10].

This case highlights the complexity of distinguishing prerenal azotemia from intrinsic renal disease in the context of acute illness. While the initial AKI was presumed secondary to dehydration from an episode of pneumonia, it could not be ruled out at that time to be a possible systemic sign of vasculitis. With the persistence of renal dysfunction and urinary abnormalities being ongoing, it was the only clue to further evaluation being necessary. Prompt diagnosis and initiation of appropriate therapy can lead to significant improvements in renal function and overall prognosis. Clinicians should maintain a high index of suspicion for AAV in patients presenting with unexplained acute kidney injury, especially when accompanied by systemic inflammatory markers and urinary abnormalities.

Early identification of pauci-immune GN is essential, as delay in treatment is associated with poor renal recovery and increased mortality [1]. Despite the presence of chronic changes on biopsy, our patient exhibited substantial improvement in renal function without requiring dialysis. This underscores the potential for renal recovery with aggressive immunosuppression, even in the setting of partially irreversible injury.

## Conclusions

Our case presentation highlights the importance of maintaining a high suspicion for AAV in patients presenting with unexplained severe worsening of kidney function along with coinciding elevated systemic inflammatory markers, in a patient with no significant past medical history. Regardless of initial assumptions of prerenal etiology, further evaluation revealed a rare but severe diagnosis of pauci-immune crescentic glomerulonephritis with medullary angiitis. Early biopsy and prompt initiation of immunosuppressive therapy, including corticosteroids, rituximab, and avacopan, resulted in improvement of renal function, with the avoidance of dialysis. This case again shows the role of timely diagnosis and aggressive treatment in improving outcomes. With confirmation from biopsy, now raises the question of the fact if the prior pneumonia was possibly a sign of systemic vasculitis, but at this time, it is harder to prove due to the prolonged time from re-admission with antibiotics, with improvement of imaging prior to arrival. 

## References

[1] Flossmann O, Berden A, de Groot K (2011). Long-term patient survival in ANCA-associated vasculitis. Ann Rheum Dis.

[2] Syed R, Rehman A, Valecha G, El-Sayegh S (2015). Pauci-immune crescentic glomerulonephritis: an ANCA-associated vasculitis. Biomed Res Int.

[3] Floyd L, Shetty A, Morris AD (2025). Clinical presentation and outcomes of antineutrophil cytoplasmic autoantibody-negative pauci-immune glomerulonephritis. Kidney Int Rep.

[4] Moiseev S, Novikov P, Jayne D, Mukhin N (2017). End-stage renal disease in ANCA-associated vasculitis. Nephrol Dial Transplant.

[5] Berden AE, Ferrario F, Hagen EC (2010). Histopathologic classification of ANCA-associated glomerulonephritis. J Am Soc Nephrol.

[6] Hendricks AR, Harris AA, Walker PD, Larsen CP (2013). Renal medullary angiitis: a case series from a single institution. Hum Pathol.

[7] Kirby G, Salas A, Alabdulsalam AK, Dasgupta A, Geetha D (2024). Clinical presentation and treatment outcomes of renal medullary angiitis in antineutrophil cytoplasmic antibody-associated vasculitis: a single-center case series. Glomerular Dis.

[8] Brix SR, Noriega M, Tennstedt P (2018). Development and validation of a renal risk score in ANCA-associated glomerulonephritis. Kidney Int.

[9] Stone JH, Merkel PA, Spiera R (2010). Rituximab versus cyclophosphamide for ANCA-associated vasculitis. N Engl J Med.

[10] Jayne DR, Merkel PA, Schall TJ, Bekker P (2021). Avacopan for the treatment of ANCA-associated vasculitis. N Engl J Med.

